# A study of leading indicators for occupational health and safety management systems in healthcare

**DOI:** 10.1186/s12913-018-3103-0

**Published:** 2018-04-23

**Authors:** Joan M Almost, Elizabeth G VanDenKerkhof, Peter Strahlendorf, Louise Caicco Tett, Joanna Noonan, Thomas Hayes, Henrietta Van hulle, Ryan Adam, Jeremy Holden, Tracy Kent-Hillis, Mike McDonald, Geneviève C. Paré, Karanjit Lachhar, Vanessa Silva e Silva

**Affiliations:** 1 School of Nursing, Queen’s University, Kingston, ON Canada; 2 School of Occupational and Public Health, Ryerson University, Toronto, ON Canada; 3 Health & Safety Professionals Inc., Sault Ste. Marie, ON Canada; 4 Kingston General Hospital, Kingston, ON Canada; 5 The Ottawa Hospital, Ottawa, ON Canada; 6 Public Services Health and Safety Association, Toronto, ON Canada; 7 Lennox and Addington County General Hospital, Napanee, ON Canada; 8 Hotel Dieu Hospital, Kingston, ON Canada; 9 Kingston Health Sciences Centre, Kingston, ON Canada

**Keywords:** Occupational health nursing, Occupational health, Leading indicators, Safety management, Management systems, Health and safety, Risk management

## Abstract

**Background:**

In Ontario, Canada, approximately $2.5 billion is spent yearly on occupational injuries in the healthcare sector. The healthcare sector has been ranked second highest for lost-time injury rates among 16 Ontario sectors since 2009 with female healthcare workers ranked the highest among all occupations for lost-time claims. There is a great deal of focus in Ontario’s occupational health and safety system on compliance and fines, however despite this increased focus, the injury statistics are not significantly improving. One of the keys to changing this trend is the development of a culture of healthy and safe workplaces including the effective utilization of leading indicators within Occupational Health and Safety Management Systems (OHSMSs). In contrast to lagging indicators, which focus on outcomes retrospectively, a leading indicator is associated with proactive activities and consists of selected OHSMSs program elements. Using leading indicators to measure health and safety has been common practice in high-risk industries; however, this shift has not occurred in healthcare. The aim of this project is to conduct a longitudinal study implementing six elements of the Ontario Safety Association for Community and Healthcare (OSACH) system identified as leading indicators and evaluating the effectiveness of this intervention on improving selected health and safety workplace indicators.

**Methods:**

A quasi-experimental longitudinal research design will be used within two Ontario acute care hospitals. The first phase of the study will focus on assessing current OHSMSs using the leading indicators, determining potential facilitators and barriers to changing current OHSMSs, and identifying the leading indicators that could be added or changed to the existing OHSMS in place. Phase I will conclude with the development of an intervention designed to support optimizing current OHSMSs in participating hospitals based on identified gaps. Phase II will pilot test and evaluate the tailored intervention.

**Discussion:**

By implementing specific elements to test leading indicators, this project will examine a novel approach to strengthening the occupational health and safety system. Results will guide healthcare organizations in setting priorities for their OHSMSs and thereby improve health and safety outcomes.

## Background

Every day, healthcare workers who strive to improve and protect the health of their patients [1] encounter physical risks from repetitive lifting and moving, chemical risks, and threats of violence [2–5]. For several years, the healthcare sector (such as hospitals, long-term care homes, retirement homes, nursing services, community care services) [6] has ranked second highest for lost-time from work injury rates among the 16 Ontario sectors in Canada, with female healthcare workers ranked the highest among all occupations for lost-time claims [7]. Approximately $2.5 billion is spent yearly on occupational injuries in Ontario’s healthcare sector [8]. This includes not only the direct costs related to premiums, benefits, surcharges and Workplace Safety and Insurance Board (WSIB) costs, but the indirect costs stemming from the long-term impact of injuries [9]. There is a great deal of focus in Ontario’s occupational health and safety system on compliance and fines. However, despite this compliance-based focus, the injury statistics are not significantly improving.

An alternative approach to changing this trend is the development of a culture of healthy and safe workplaces, including the effective utilization of leading indicators within Occupational Health and Safety Management Systems (OHSMSs). In Ontario, the Ontario Safety Association for Community and Healthcare (OSACH) designed an OHSMS to assist healthcare organizations to advance and link a culture of health, safety and wellness with employee safety and wellness. While it is recognized all 82 elements of the OSACH system are important, availability of resources limit the ability of organizations to address all of them. Therefore, the purpose of this study is to conduct a longitudinal study implementing six elements of the OSACH system identified as leading indicators and evaluating the effectiveness of this intervention on improving selected health and safety workplace indicators. The leading indicators used in this study were identified in an industrial model proposed by Bennett and Foster [10]. The elements include senior management commitment, continuous improvement, communication, competence, employee involvement in occupational health and safety, and occupational health management [10]. By implementing specific elements to test leading indicators, this project will examine a new approach to strengthening the occupational health and safety system.

### Occupational health and safety management systems

OHSMSs are a combination of planning and review, consultative arrangements, and specific program elements that work together in an integrated way to improve health and safety performance [11]. OHSMSs are distinguishable from traditional occupational health and safety (OHS) programs by being more proactive, better integrated internally and incorporating elements of evaluation and continuous improvement [12]. Organizations that adopt an OHSMS have a clear vision of health and safety goals, communicate these goals to their workforce, assess risk data, define corrective action more often, and exhibit improved attitudes towards employee training [13]. Research has linked low injury rates in organizations with elements of OHSMSs [14–17]. However, in a systematic review examining OHSMSs, Robson et al. [12] identified a number of gaps in the research, more specifically a lack of rigorous research examining the effectiveness of OHSMSs interventions on employee health and safety outcomes. This gap will be addressed in this proposed project by developing and evaluating the effectiveness of a tailored intervention on improving health and safety workplace indicators.

### Leading indicators

Many organizations put programs in place, but do not develop an overall OHSMS. When an organization is focused on a program, lagging indicators are often used to measure the outputs of the program [18]. A lagging indicator is defined as a measure taken after events with a focus on outcomes and occurrences based on retrospective data [19]. Lagging indicators include accident and incident rates, disease statistics, frequency of accident investigations and costs associated with compensation systems [18, 20, 21]. Focusing on lagging indicators does not lead to sustainability or evaluate the quality of an OHSMS. Thus, there has been a movement away from lagging indicators, towards ‘leading’ or predictive assessments of an organization’s health and safety climate. A leading indicator is often associated with proactive activities and is a condition or measure that precedes an event. By identifying and using leading indicators, performance outcomes under current conditions of an organizational system may be anticipated and action taken.

Much has been written about leading indicators for OHSMSs, but there is inconsistent terminology, lack of uniformity, and little discussion on how to measure these indicators. The template of leading indicators identified by Bennett and Foster [10] was chosen for this project because the indicators are well-developed, measurable and based on an extensive literature review of other industries. *Senior management commitment* is about more than a policy being written and posted on a safety board. Health and safety goals must be ingrained in organizations with health and safety objectives required at the senior management level and included in all team-meeting agendas [10]. *Continuous improvement* within OHSMSs is never stagnant. Health and safety is an ongoing process that needs to change and grow with the employer being committed to improving the system. Managers set and review objectives which are integrated into operational management [10]. The committed organization has a process in place that implements audits, completes investigations, and deals with non-conformities quickly. It is not enough to keep track of incidents but instead understand how the system failed, and what led to the incident. *Communication* includes interactive discussions as well as company-wide messages supportive of OHS from a senior person within the organization. Employee communication and feedback are paramount to improved OHS [18, 22]. *Competence* includes an analysis of all jobs for competence requirements including OHS understanding. The front-end hiring and training of new personnel is the most effective step in the consideration of health and safety performance [23]. The expectation is that workers have received health and safety induction, and an individual has confirmed qualifications for the work being undertaken. *Employee involvement* focuses on the involvement of each employee in an OHS activity rather than the expectation that the health and safety department is solely responsible for OHS. This can include workplace inspections, incident investigations, or working on risk assessments. The goal is to stimulate everyone within the organization to take responsibility and understand various aspects of OHS. *Occupational health management* includes the monitoring of occupational health risks such as violence, musculoskeletal disorders, and infectious diseases.

## Methods

### Aim

To conduct a longitudinal study implementing six elements of the OSACH system identified as leading indicators and evaluating the effectiveness of this intervention on improving selected health and safety workplace indicators.

### Research questions

1. What are the current OHSMSs and leading indicators being used by participant sites?
2. What are the facilitators and barriers to changing current OHSMSs in participant sites?
3. Which of the six leading indicators could be added or changed at each site?
4. Is the tailored OHSMS intervention using the six leading indicators effective in improving health and safety workplace indicators over one year, specifically absenteeism, workers’ compensation composite, training and professional development opportunities, manager/supervisor training, and health and safety climate?

### Design

A quasi-experimental longitudinal research design will be used. There will be two phases of the project. The first phase will focus on assessing current OHSMSs in participating hospitals using the key leading indicators, determining potential facilitators and barriers to changing current OHSMSs, and identifying the leading indicators that could be added or changed to the existing OHSMS in place. Phase I will conclude with the development of an intervention designed to support optimizing current OHSMSs in participating hospitals based on identified gaps. Phase II will pilot test and evaluate the tailored intervention.

### Setting

The setting will include two acute care hospitals in Eastern Ontario. Sites will be chosen based on size (small, medium, large) and location (urban and rural) to obtain a variation in types of hospital.

### Phase I assessment and development

Data collection in phase I will include 1) semi-structured interviews and document reviews; 2) surveys; and, 3) administrative data.

#### Semi-structured interviews and reviewing of hospitals documents

To answer research questions #1 and #2, semi-structured interviews will be used to assess the current OHSMS and leading indicators being used, and determine potential facilitators and barriers in the participating hospitals. The information obtained from the interviews will also identify gaps and areas for improvement (research question #3) to be addressed in phase II. ***Sample*:** The Chief Executive Officer (CEO), members of the administrative team (e.g. Vice-Presidents, Directors) and members of the Occupational Health and Safety Department and/or the Joint Health and Safety Committee will be invited to participate. If interview participants are unable to provide the necessary information, they will be asked to provide the name(s) of the most appropriate person to contact. ***Interview Questions:*** An interview guide will be developed. Interview questions to assess key leading indicators will be developed using the template by Bennet and Foster (2005) and the corresponding elements found in the OSACH (2009) Assessment Tool. Based on the answers and documentation provided by each site, each leading indicator will be given a rating of 0 (never completed) to 10 (100% completed) to ensure consistency. A similar categorization was used by Bennett and Foster [10] to identify areas of potential improvement and to allow comparison to data collected in phase II. The objective is full implementation of each indicator; therefore, a score of 10 indicates no improvement is required.

***Data collection:*** Each interview will take place over 1 week in order to complete the preliminary assessments with two research assistants (RA) (one team of one consultant and one RA for each site). Each team will conduct the interviews together to ensure no important cues or interpretations are missed. In order to ensure consistency during the assessment, a meeting will take place with the two teams and the PI to review the questions and ensure a clear understanding of what data is required. The consultant will facilitate the interview and the RA will take notes. The RA will also be trained for their role during the intervention phase. Participants will be asked to provide documentation to support their answers, such as meeting minutes, emails, etc. Document review will be recorded using a standard form to ensure consistent measurement. Interviews will be audio-recorded should the consultant and RA need to review the recording for missing information or clarification. Transcription will not be required.

### Surveys

To evaluate the effectiveness of the tailored intervention (research question #4), survey data will be collected at the beginning of phase I, and near the end of phase II using an online survey. ***Sample:*** A power analysis was conducted using G*Power [24] to determine the appropriate sample size. A paired t-test will be used to assess for statistical significance in the outcome variables pre- and post- intervention. For the paired t-test statistic with an alpha significance level of .05 and a power level of .95, it was determined that a moderate effect size (*r* = .50) between groups would be detected with a total sample size of 45 participants. Each site will be assessed separately. Therefore, 45 participants are required at each site with a total of 90 required participants. To compensate for a 50% response rate commonly found in survey research, a minimum of 180 participants is recommended. The number of employees in each hospital identified in the region varies with a range from 270 to 4000. Therefore, to ensure the appropriate sample size obtained, all employees (full-time, part-time and casual) and physicians in the participating sites will be invited to participate. Hospital volunteers will be excluded.

#### Instruments

While health and safety culture is the product of values, beliefs, competencies and patterns of behavior that determine the commitment to an organization’s health and safety [25], the health and safety climate is the result of that higher level health and safety culture [26]. The Health and Safety Climate Assessment Tool was developed through a joint industry and United Kingdom Health and Safety Executive research project to assess health and safety culture in offshore environments. The Public Services Health and Safety Association (PSHSA) (formerly OSACH) implemented a modified version of the tool [9] at four Ontario healthcare organizations as a means to improve health and safety outcomes. The assessment tool is organized based on three sections: Attitude Assessment and Questionnaires, Focus Groups and Interviews and/or Surveys and Behaviour and Observational Assessment. In this project, only the first section, Attitude Assessment and Questionnaires, will be used. Participants are asked to respond to 43 questions on a 5-point Likert scale from strongly agree to strongly disagree. The questions inform the results for nine dimensions: 1) management commitment, 2) communication, 3) priority for safety, 4) safety rules and procedures, 5) supportive environment, 6) involvement, 7) personal priorities and need for safety, 8) personal appreciation of risk, and 9) physical work environment. A demographic questionnaire will include questions about the participant’s age, years of experience in current role, years of experience at hospital, education, and employment status (full-time, part-time, casual).

#### Data collection

Each participant will receive an email with an URL link to an online survey using Fluid Survey. An individual identification code will be provided to enable follow-up with non-respondents and linking data for analysis from the pre- and post-intervention data collection as a means to evaluate the effectiveness of the intervention. An introductory screen will briefly describe the survey, project purpose, participant confidentiality, and researcher contact information. As suggested by Dillman et al. [27], a follow-up reminder email will be sent to non-respondents 12 days after the initial email, followed by a final email reminder two weeks later.

#### Data analysis

Descriptive statistics will be used to summarize the characteristics of participants. Means and standard deviations will be calculated for each of the dimensions. An algorithm will be used to calculate a score out of ten for each of the nine dimensions, zero being the worst score and ten being the best score. These scores will then be plotted on a graph representing an overall ‘snapshot’ of the organization’s current health and safety climate. Reliability analysis using Cronbach’s alpha will be conducted on the multi-item scales to determine the reliability of the measurement tools in this sample.

### Administrative data

To evaluate the effectiveness of the tailored intervention (research question #4), administrative data will be collected at the beginning of phase I, and near the end of phase II. In a recent project, PSHSA [19] recommended a set of core, consensus-based health and safety workplace indicators. From this set, the following administrative data will be collected:

#### Absenteeism [19]

Participant hospitals will be asked to provide the following data on absenteeism: Eligible employee groups defined as employees eligible for sick time benefits (e.g. not casual or contract staff). Sick leave hours include paid time for sick leave (i.e. absence due to medical leave, not other reasons such as family emergency) up until 15 weeks (75 days). Regular paid hours are defined as the hours regularly worked by the employee group. The following calculation will be used to determine the percentage of sick hours (absenteeism): Percentage sick hours = (Sick Leave Hours for all eligible employee groups / Regular paid hours for all eligible employee groups) × 100.

#### Workers compensation composite [19]

Written permission will be obtained by the Principal Investigator from participant hospitals prior to requesting the following data from WSIB to create a composite index measure:

- Average lost-time claims accepted per 100 FTEs (200,000 h) annually (frequency)
- Average number of days lost per 100 FTEs (200,000 h) annually (severity)
- Number of lost-time claims accepted per 100 FTEs (200,000 h) annually (frequency)
- Number of health care no lost-time (NLT) claims accepted per 100 FTEs (200,000 h) annually (frequency)
- Number of days lost per 100 FTEs (200,000 h) annually (severity)
- Number of injuries for injury categories over the previous year. These categories include sprains and strains, bruises and contusions, fractures, traumatic injuries, disorders, and complications, multiple traumatic injuries, fall on same level, overexertion, bodily reaction, assaults, violent acts, or harassment, struck by an object or equipment.

#### Training & Professional Development Opportunity [19]

Participant hospitals will be asked to provide data on training and professional opportunity hours and total number of eligible employees.

#### Manager/supervisor training [19]

Participant hospitals will also be asked to provide data on total number of managers and supervisors and the number trained in due diligence.

### Analysis of phase I

Following the completion and analysis of the interviews, document review, administrative data and survey results, an all-day face-to-face meeting will be held with the research team and participating sites. The purpose will be to review the results, identify gaps, identify which leading indicators need to be changed or added, and develop the tailored intervention for each site. Ultimately the objective is the implementation of all six leading indicators. However, it may not be realistic to address several missing indicators, given the time frame of this project. The research team, in collaboration with the OHS director/manager, will determine the indicators which could be improved, and how to address the gaps identified. A teleconference will take place with the research team, PSHSA, and knowledge users within two weeks of the intervention being implemented to finalize the plan.

### Phase II intervention

The tailored intervention will be implemented and monitored over 12 months. The internal work will be completed by appropriate individuals within each site, while our team of consultants and RAs will provide support in the implementation. The implementation and monitoring will initially be a joint effort between the consultants and RAs. This initial monitoring time will also be used by the consultant to equip the RA with the necessary knowledge to monitor the intervention. All subsequent monitoring will be the responsibility of the RA. The intervention will deliver various elements of the leading indicators to each participating hospital; however, the elements will vary depending on the identified gaps, facilitators and barriers in phase I. The goal of the intervention will be to improve OHSMS in each hospital to meet the specific quantified benchmarks based on the assessment questions from phase I. Each RA will meet with the personnel responsible for implementation in each participant site monthly, answer questions, and ensure the intervention is being implemented as planned. Similar to phase I, participants will be asked to provide copies of documentation to illustrate examples of each indicator. The hospital documents will be reviewed by the RA and recorded using the same standard form to ensure consistent measurement. Similar to phase I, each indicator will be assessed at the end of the intervention with a rating of 0 (never completed) to 10 (100% completed) to compare to the rating in phase I. The data to assess each indicator will be collected over 12 months. During the 12-month intervention, teleconference meetings will take place with the researchers, PSHSA, and knowledge users every 5 months (e.g. 11th and 16th month) to provide updates on the progress of the project and discuss any issues that arise. At the end of the intervention (19th month), a teleconference meeting will take place between researchers, PSHSA, knowledge users, and collaborators to discuss the intervention overall and confirm questions for the interviews, document review, surveys and administrative data being collected to assess the intervention.

### Approach to evaluation of intervention in phase II

#### Interviews

Semi-structured interviews will be used to assess the implementation process and tailored intervention. *Sample*: The CEO and Director/Manager of the Occupational Health and Safety Department will be invited to participate. *Data collection:* Each interview will be approximately 1.5 h in length and will be conducted by the project PI and RA to ensure no important cues or interpretations are missed. The RA will take notes. The interview guide will be followed. Interviews will be tape-recorded to allow the PI and RA the opportunity to review the recording for missed information or clarification. Transcription will not be required.

#### Surveys

In order to assess the effectiveness of the intervention (research question #4), data collection from phase I will be repeated at the end of the intervention. *Sample:* Surveys will be distributed to all employees to assess for overall changes pre- and post-intervention. For participants who completed surveys in both time points, data will be linked in order to assess change over time. *Data analysis*: Descriptive statistics will be used to summarize the characteristics of participants. Means and standard deviations will be calculated for each dimension. An algorithm will be used to calculate a score for each of the nine dimensions, zero being the worst score and ten being the best score. These scores will then be plotted on a graph presenting an overall ‘snapshot’ of the organization’s current health and safety climate. Reliability analysis, using Cronbach’s alpha, will be conducted on the multi-item scales to determine the reliability of the measurement tools in this sample. Frequencies and percentages will be used to summarize the evaluation of the OHSMS. Paired t-tests will be used to test for significant changes in each variable measured with participants who completed surveys at both time periods. Unpaired t-tests will also be conducted on the pre- and post-intervention data to allow for the inclusion of data from individuals who only participated in one data collection period.

#### End of phase II

Following the completion and analysis of the interviews, document review, administrative data and survey, a final teleconference will take place with the entire team one month prior to the final symposium to review the results, discuss recommendations, and prepare the final report. A final one-day symposium will be held with all team members in the final month. Knowledge users who were not part of the project (e.g. non-participant hospitals and healthcare organizations) and policy makers (e.g. representatives from Ministry of Labour (MOL), Ministry of Health and Long-term Care (MOHLTC), Ontario Hospital Association (OHA)) will be invited to the one-day symposium. The purpose of the symposium will be to present the project results and ensure the results are made available to all healthcare organizations and future government initiatives.

## Discussion

Most occupational health and safety professionals agree there is a need for OHSMSs, however, the available research is weak and there is a gap in our knowledge. Although there are many similarities among all OHSMS elements, there is no agreement on which of these elements is most beneficial to workplace health and safety. Our proposed research expands on work done in Ontario by OSACH. This research project endeavors to focus on six key leading indicators of an OHSMS and show that these key indicators can have a positive effect on a workplace health and safety culture, and ultimately reduce injury and illness in the workplace. If shown to have an impact, the straightforward actions of a CEO to get out in front of the workers, and become the workplace champion can be a very simple, cost effective, yet powerful action to make health and safety changes within an organization. With money at a premium in the healthcare system, finding ways to save money, while improving the efficiency of an organization, is imperative.

### Potential impact and future directions

There is a great deal of focus in Ontario’s occupational health and safety system on compliance and fines. Occupational health and safety professionals believe focusing on managed systems and a health and safety culture within an organization, in most cases, is a more effective alternative to fines. This research proposes to give healthcare organizations an alternative to a compliance-based system.

Over the next decade, the healthcare sector faces several challenges which could have a significant impact on worker health and lost-time injury rates. These challenges include increased care requirements resulting from Ontario’s aging population, increased demand on health and community care services, and globalization of occupational health and safety issues such as emerging infectious diseases, pandemics and other environmental risks. Across Ontario, there are competing priorities faced by healthcare organizations with time and money at a premium. Enhancing a culture of healthy and safe workplaces across Ontario’s healthcare system is essential.

## Abbreviations

CEO: Chief executive officer
FTE: Full-time employee
MOHLTC: Ministry of Health and Long-term Care
NLT: Number of lost-time
OHS: Occupational health and safety
OHSMS: Occupational health and safety management systems
OSACH: Ontario Safety Association for Community and Healthcare
PI: Principal investigator
PSHSA: Public Services Health & Safety Association
RA: Research assistant
WSIB: Workplace safety and insurance board
